# Suicide attempt and its associated factors amongst women who were pregnant as adolescents in Bangladesh: a cross-sectional study

**DOI:** 10.1186/s12978-021-01127-6

**Published:** 2021-03-31

**Authors:** Jie Li, Syeda Zerin Imam, Zhengyue Jing, Yi Wang, Chengchao Zhou

**Affiliations:** 1 Centre for Health Management and Policy Research, School of Public Health, Cheeloo College of Medicine, Shandong University, Jinan, Shandong China; 2 NHC Key Laboratory of Health Economics and Policy Research, Shandong University, Jinan, Shandong China

**Keywords:** Suicide, Suicide attempt, Adolescent, Pregnancy, Health status, Social support

## Abstract

**Background:**

Adolescent pregnancy is a risk factor for suicide. We aimed to assess the prevalence of suicide attempts among young women with adolescent pregnancy in Bangladesh and to explore its associated factors.

**Methods:**

In this cross-sectional study, we surveyed young women with adolescent pregnancy in urban and rural areas in Bangladesh to assess suicide attempts, socio-demographic and pregnancy-related characteristics, perceived health status, and perceived social support. Binary logistic regression analysis was conducted to explore the relationship between potentially related factors and suicide attempts.

**Results:**

Of the participants, 6.5% (61/940) reported suicide attempts in the past 12 months, and the majority (88.5%) of the attempts happened within one year after the pregnancy. Participants with more years after first pregnancy (odds ratio (OR) = 0.47, 95% CI: 0.37–0.61) and more perceived social support from friends (OR = 0.69, 95% CI: 0.55–0.86) were less likely to have suicide attempts, and those perceived bad health status compared with good/fair health status (OR = 8.38, 95% CI: 3.08–22.76) were more likely to attempt suicide.

**Conclusions:**

Women with adolescent pregnancy were at high risk of suicide attempts, especially those during the first postnatal year. The risk of suicide attempts attenuated with the time after pregnancy, and perceived social support from friends was a protective factor and perceived bad health status was a risk factor for suicide attempts among young women who have experienced adolescent pregnancy.

## Plain English summary

Adolescent pregnancy is a major public health problem around the world, and it was a risk factor for suicide. It occurs in high, middle, and low-income countries, with higher prevalence in low-income countries (e.g. Bangladesh). In this study, young women with experiences of adolescent pregnancy were investigated for the prevalence of suicide attempts and its association with socio-demographic and pregnancy-related factors, health status, and social support.

Participants were recruited from women who presented to the selected departments of gynecology and obstetrics and maternal health care centers in Bangladesh. They were interviewed to complete questionnaires including their socio-demographic characteristics, first-pregnancy-related factors, self-reported health status, perceived social support, and suicide attempts.

Of the 940 participants, 61 (6.5%) reported suicide attempts in the past 12 months. The majority (88.5%) of the attempts happened within one year after the pregnancy. Participants with one more year after first pregnancy were 53% less likely to have suicide attempts, those with one more point score of perceived social support from friends were 31% less likely to have suicide attempts, and those perceived bad health status compared with good/fair health status were 7.38 times more likely to attempt suicide.

In conclusion, young women with adolescent pregnancy were at high risk for suicide attempts in Bangladesh, especially those with shorter time after adolescent pregnancy and perceived bad health condition. Improving friends’ support may help to protect these women from suicide attempts.

## Background

Adolescent pregnancy, defined as pregnancy between ages 10–19 years, is a major public health problem around the world. It occurs in high, middle, and low-income countries, with the highest prevalence in low-income countries (e.g. Bangladesh) [1]. Globally, an estimated 21 million girls aged 15–19 years become pregnant in 2016 in developing regions [2], and it increases especially in Asia and Africa [3]. Bangladesh is a low-income country located in South Asia. It has the highest adolescent fertility rate in South Asia, with an estimated rate of 83.5 per 1000 girls aged 15–19 years every year between 2015 and 2020 [4].

Adolescent pregnancy may compromise the educational and occupational opportunities of the young mother. It also harms their psychological health, even increases the risk of suicide [5, 6]. Meanwhile, females were found to be more likely to attempt suicide than males [7–10]. The experience of suicide attempts increased the risk of subsequent attempts and completed suicide [11]. Since females are at a higher risk of suicide attempts and pregnancy further increased the risk, it is necessary to explore suicide attempts among young women with adolescent pregnancy. However, the prevalence of suicide attempts in this special population remains unclear.

Clarifying the related factors is important in preventing suicide among young women who have experienced adolescent pregnancy [12]. Some socio-demographic characteristics, such as age, education, location, employment status, and health status were found to be related to suicide in the general population and veterans [9, 13–16]. Pregnancy-related factors (e.g. unplanned pregnancy and outcomes of pregnancy) were found to be associated with elevated maternal depression in general maternal women, which were dominant risk factors for suicide attempts in women [17–19]. However, the relationships between these factors and suicide attempts among women with adolescent pregnancy have been less studied. Besides, social support was found to be able to buffer individuals from suicidal ideation [15, 20]. The relationship between each dimension of social support, including supports from families, friends, and significant others, and suicide needs further investigation in this vulnerable population.

Although women with adolescent pregnancy were evidenced to be at risk of suicide, there is a paucity of reports on prevalence among this special population. Since the experience of suicide attempts was a strong predictor of future suicidal behavior, it is important to explore its associated factors. Thus, this study was conducted to assess the prevalence of suicide attempts among young women with adolescent pregnancy and to explore its association with socio-demographic and pregnancy-related factors, health status, and social support.

## Methods

### Study design and settings

A cross-sectional survey was conducted from December 2018 to February 2019 in Bangladesh. Five hospitals from the urban area (Bangabandhu Sheikh Mujib Medical University, Dhaka Medical College, Marie Stopes Clinic and Maternity, Shaheed Suhrawardy Medical College, and Community Hospital Dhaka) and five rural places (Savar, Narayangonj, Gazipur, Mymensing, and Tangail) have been selected depending on the availability of the patients. The departments of gynecology and obstetrics from the five hospitals in Dhaka and five maternal health care centers in those rural areas were selected as the settings.

### Participants

Participants of the study were recruited from all those women who presented to the selected departments of gynecology and obstetrics and maternal health care centers from December 2018 to February 2019. The inclusion criteria were that (1) first pregnancy at the age of 17 years old or younger because we wanted to focus on the women who were pregnant before legal marriage age of 18 years old in Bangladesh, (2) without severe medical conditions which may increase the risk of suicide, such as cancer, heart failure, brain injury, and psychiatric disorders, and (3) willingness to participate in the survey. Women who were unable to complete the survey because of the limited ability of communication or others were excluded.

### Study procedure

The survey was administered by field workers. They were trained before the survey and supervised during data collection. Data were collected through face-to-face interviews after obtaining the informed consent of the participants. Respondents were asked about their socio-demographic characteristics (e.g., age, marital status, education, place of birth, and occupation) and first-pregnancy-related factors (e.g., age, outcome, plan status, and marital status of the first pregnancy). In addition, the respondents were asked about the self-reported health status, suicide attempts, and perceived social support.

### Measures

#### Personal factors (socio-demographic and pregnancy-related characteristics and health status)

Information on women’s socio-demographic and first-pregnancy-related characteristics, such as age, marital status, education, age of the first pregnancy, planned or unplanned pregnancy, and reason and outcome of the first pregnancy, were obtained through the questions designed by researchers based on the purpose of the survey. Perceived health status was a self-reported subjective measurement of general health. It has been shown to be a sensitive indicator of overall health [21]. A single item was used to ask the participants to rate their health condition on a five-point Likert scale (1 = “very good”, 2 = “good”, 3 = “fair”, 4 = “bad”, and 5 = “very bad”) to assess the perceived health status in the current study [21].

#### Suicide attempts

We used the question “Have you ever tried to kill yourself in the past 12 months?” from the Ask Suicide-Screening Questions Toolkit [22] to assess the experience of suicide attempts in the past 12 months prior to the assessment with the answer of “yes” or “no”.

#### Perceived social support

The Perceived Social Support Scale (PSSS) [23] was used to assess the social support of the participants. It contains 12 items scored on a seven-point Likert scale which is divided into three subscales: family (4 items), friends (4 items), and significant others (4 items). The total subscale score is calculated by adding the item score for each subscale. A higher score indicates a higher level of perceived social support. The Cronhach’s alpha values of the total scale and subscales of family, friends, and significant others were 0.88, 0.90, 0.77, and 0.93 in this study, respectively.

### Statistical analysis

Independent sample *t*-tests (continuous variables) and Chi-square tests (categorical variables) were used to compare differences between participants with and without suicide attempts. The responses of perceived health status were categorized into two groups in the analyses: a group of good/fair health from the original responses 1 = “very good”, 2 = “good”, and 3 = “fair” and a group of bad health from the original responses 4 = “bad” and 5 = “very bad”. A binary logistic regression analysis was conducted to explore the relationship of socio-demographic variables, pregnancy-related variables, perceived health status, and social support with suicide attempts. We obtained the odds ratio (OR) and its 95% confidence interval (CI) from the logistic regression analysis. The factors that showed possibly significant associations (at the *p*-value of 0.1) with suicide attempts in independent sample *t*-tests and Chi-square tests were included in the multivariate analyses as independent variables. As years after first pregnancy were computed by age minus age of first pregnancy, they were analyzed separately in logistic regression models to avoid collinearity. Two-sided *p*-values of 0.05 were considered as the significance level. Statistical analyses were performed using SPSS 24.0 (IBM Corp., Armonk, NY, USA).

## Results

### Sample characteristics

Of the 2,500 participants we approached, 983 were eligible and agreed to participate, and 940 (95.6%) completed the questionnaires with valid responses. Table 1 shows the participants’ socio-demographic and pregnancy-related characteristics. The mean age of the participants was 23.17 years (SD = 4.05) with a range from 15 to 34 years old. 783 (83.3%) participants were married and 661 (70.3%) participants reported that their health condition was good or fair. There were four (0.4%) participants who experienced first pregnancy at the age of 14 years old and 284 (30.2%) at the age of 15 years old, 448 (47.7%) at the age of 16 years old, and 204 (21.7%) at the age of 17 years old. The mean years after the first pregnancy was 7.26 (SD = 4.18), ranging from 0 to 19 years. The percentage of participants who terminated their first pregnancy was 12.9% and who were pregnant without a plan was 56.7%. Of the whole 940 participants, 61 (6.5%) participants reported suicide attempts in the past 12 months.

**Table 1:** Socio-demographic and pregnancy-related characteristics of the participants (N = 940)

	n	%
Age, mean ± SD, years	23.17 ± 4.05	
15–19	163	17.3
20–34	777	82.7
Marital status		
Single	157	16.7
Married	783	83.3
Education		
Illiterate/semi-literate/primary school	690	73.4
High school and college	250	26.6
Place of birth		
Rural	857	91.2
Urban	83	8.8
Current working status		
Unemployed	487	51.8
Employed	453	48.2
Dropout experience from the school		
No	135	14.4
Yes	805	85.6
Perceived health status		
Good/fair	661	70.3
Bad	279	29.7
Age of the first pregnancy, years		
14–15^a^	288	30.6
16	448	47.7
17	204	21.7
Years after the first pregnancy		
Within 1 year	79	8.4
More than 1 year	861	91.6
Outcomes of the first pregnancy		
Completed or carrying	819	87.1
Terminated	121	12.9
Pregnant plan of the first pregnancy		
No	533	56.7
Yes	407	43.3
Reasons of first pregnancy		
Normal	925	98.4
Abnormal (e.g., rape)	15	1.6
Suicide attempt		
No	879	93.5
Yes	61	6.5

^a^There were four participants (0.4%) reported their first pregnancy at the age of 14 years

### Differences between participants with and without suicide attempts

**Fig. 1:**
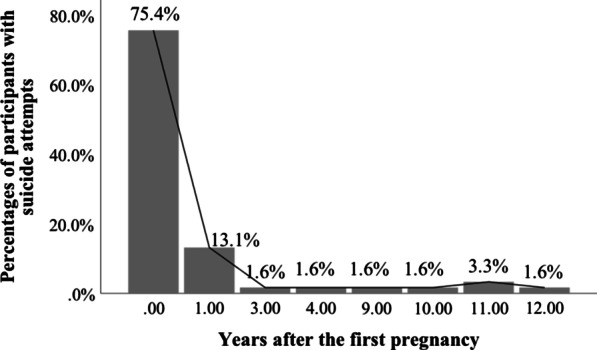
Percentages of participants with suicide attempts in different years after the first pregnancy

Participants with suicide attempts were significantly younger than those without suicide attempts. More participants were unemployed and perceived bad health status in females with suicide attempts than females without suicide attempts. The distributions of the age of first pregnancy were significantly different between the two groups; the percentage of women who were pregnant for the first time at the age of 17 years old in participants with suicide attempts was higher than those without suicide attempts. Years after the first pregnancy were significantly less among participants with suicide attempts than those without. Of the 61 females with suicide attempts, 88.5% (54/61) were within one year after the pregnancy, which was significantly higher than those among females without suicide attempts (2.8%, 25/879; *χ*^2^ = 543.98, *p* < 0.001). The distribution of women attempting suicide in different years after the first pregnancy is shown in Fig. 1. Perceived social support from family, friends, and significant others was significantly less in participants with suicide attempts than those without. The percentage of participants who terminated their first pregnancy and the percentage of participants whose pregnancies were planned were higher among women with suicide attempts than those without. There were no significant differences in the distributions of marital status, education, place of birth, dropout experience from the school, and reasons of first pregnancy between participants with and without suicide attempts (See Table 2).

**Table 2:** Comparisons of the potential associated factors between participants with and without suicide attempts (N = 940)

	With suicide attempts (n = 61)	Without suicide attempts (n = 879)	*t/χ*^2^	*p*
Age, mean ± SD, years	17.57 ± 2.84	23.56 ± 3.83	15.49	< 0.001
Marital status			1.00	0.318
Single	13 (21.3)	144 (16.4)		
Married	48 (78.7)	735 (83.6)		
Education			0.05	0.816
Illiterate/semi-literate/primary school	44 (72.1)	646 (73.5)		
High school and college	17 (27.9)	233 (26.5)		
Place of birth			2.84	0.092
Rural	52 (85.2)	805 (91.6)		
Urban	9 (14.8)	74 (8.4)		
Current work status			35.22	< 0.001
Unemployed	54 (88.5)	433 (49.3)		
Employed	7 (11.5)	446 (50.7)		
Dropout experience from the school			0.01	0.928
No	9 (14.8)	126 (14.3)		
Yes	52 (85.2)	753 (85.7)		
Perceived health status			70.13	< 0.001
Good/fair	14 (23.0)	647 (73.6)		
Bad	47 (77.0)	232 (26.4)		
Age of first pregnancy, years			63.35	< 0.001
14–15	10 (16.4)	278 (31.6)		
16	13 (21.3)	435 (49.5)		
17	38 (62.3)	166 (18.9)		
Years after first pregnancy			543.98	< 0.001
Within 1 year	54 (88.5)	25 (2.8)		
More than 1 year	7 (11.5)	854 (97.2)		
Outcomes of the first pregnancy			35.87	< 0.001
Completed or carrying	38 (62.3)	781 (88.9)		
Terminated	23 (37.7)	98 (11.1)		
Pregnant plan of the first pregnancy			15.20	< 0.001
No	20 (32.8)	513 (58.4)		
Yes	41 (67.2)	366 (41.6)		
Reasons of first pregnancy			4.59	0.068^a^
Normal	58 (95.1)	867 (98.6)		
Abnormal (e.g., rape)	3 (4.9)	12 (1.4)		
Perceived social support				
Family	8.89 ± 3.87	13.58 ± 3.06	9.27	< 0.001
Friends	11.93 ± 2.64	14.51 ± 2.40	7.39	< 0.001
Significant others	8.46 ± 6.73	12.16 ± 5.20	4.21	< 0.001

^a^Fisher’s exact test was used

### Associated factors of suicide attempts

We firstly performed logistic regression by including age, age of first pregnancy, and other variables except for years after the first pregnancy with the *p*-values of 0.1 or less in univariate associations as independent variables. The results are presented in Table 3. Women who were older (OR = 0.46, 95%: CI 0.35–0.62) and perceived more social support from friends (OR = 0.68, 95% CI: 0.54–0.85) were less likely to have suicide attempts. Women who perceived bad health status compared with those perceived good/fair health (OR = 8.91, 95%: CI 3.14–25.30) and who were pregnant for the first time at 17 years old compared with those at 14–15 years (OR = 3.67, 95%: CI 1.05–12.82) were more likely to have suicide attempts.

**Table 3:** Multivariate associations between demographic characteristics, pregnancy-related characteristics, health status, and perceived social support and suicide attempts (N = 940)

	*B*	*S.E*	*Wald χ*^*2*^	*p*	OR (95%CI)
Age	− 0.77	0.15	27.41	< 0.001	0.46 (0.35–0.62)
Place of birth					
Rural	Reference				1
Urban	0.98	0.86	1.31	0.253	2.67 (0.50–14. 38)
Current work status					
Unemployed	Reference				1
Employed	0.89	0.78	1.31	0.253	2.44 (0.53–11.25)
Perceived health status					
Good/fair	Reference				1
Bad	2.19	0.53	16.90	< 0.001	8.91 (3.14–25.30)
Age of first pregnancy, years					
14–15	Reference				1
16	1.01	0.65	2.44	0.118	2.75 (0.77–9.73)
17	1.30	0.64	4.14	0.042	3.67 (1.05–12.82)
Outcomes of the first pregnancy					
Completed or carrying	Reference				1
Terminated	0.43	0.51	0.72	0.396	1.54 (0.57–4.14)
Pregnant plan					
No	Reference				1
Yes	− 1.04	0.65	2.59	0.107	0.35 (0.10–1.25)
Reasons of first pregnancy					
Normal	Reference				
Abnormal (e.g., rape)	− 0.07	0.09	0.71	0.400	0.93 (0.79–1.10)
Perceived social support					
Family	− 0.07	0.09	0.71	0.400	0.93 (0.79–1.10)
Friends	− 0.39	0.11	11.59	0.001	0.68 (0.54–0.85)
Others	0.04	0.07	0.29	0.590	1.04 (0.91–1.19)

Dependent variable: suicide attempts (0 = without, 1 = with)

We then performed logistic regression by including years after first pregnancy instead of age and age of first pregnancy. The results show that participants with more years after first pregnancy (OR = 0.47, 95%: CI 0.37–0.61) and more perceived social support from friends (OR = 0.69, 95% CI: 0.55–0.86) were less likely to have suicide attempts, and those perceived bad health status (OR = 8.38, 95% CI: 3.08–22.76) were more likely to have suicide attempts in the past 12 months.

## Discussion

The current study is the first to examine the prevalence of suicide attempts and their relationship with socio-demographic and pregnancy-related characteristics, perceived health status, and perceived social support in young women who were pregnant for the first time in adolescence. The results show that the past-year prevalence of suicide attempts was 6.5% in the whole sample, and the majority (88.5%) of the attempts happened within one year after the pregnancy. Those who were younger, pregnant for the first time at a relatively older age, perceived bad health status, and reported less perceived social support from friends were more likely to commit suicide in the past 12 months of the survey.

A meta-analysis including 88, 225 college students in China found that the prevalence of 12-month suicide attempts was 2.9% and the prevalence of suicide attempts for female students was 2.7% [10]. Meehan et al. [24] reported the prevalence of 12-month suicide attempts was 2.0% among young adults in a public university in the US. The prevalence of suicide attempts in young women with adolescent pregnancy found in our study (6.5%) was higher than that in the general population of a similar age. There are few reports of the prevalence of suicide attempts in this age group in Bangladesh. One study found that the prevalence of suicide attempts in adults aged ≤ 39 years from the rural locations was 0.3% [25]. Another study on suicidal ideation reported that the prevalence of suicidal ideation among women aged 15 to 49 years ranged from 11.0 to 14.0% [26], however, less than one-third of them will commit suicide attempts [27]. In conclusion, young women with adolescent pregnancy in Bangladesh reported a relatively higher prevalence of suicide attempts than the general population at a similar age.

The current study found that among women who have experienced adolescent pregnancy, those with older age were less likely to commit suicide. This may be because the effect of pregnancy happened in their period of adolescence on psychological well-being was attenuated over time. Our further analysis indicated that women with a longer period after the adolescent pregnancy were less likely to attempt suicide, confirming the possible reason. Women who were within one year after the pregnancy accounted for 88.5% of all the women attempting suicide in this study. One possible reason for the higher suicide risk of a shorter time after pregnancy was that adolescents were vulnerable in this rapid phase and critical period for human development [28], and pregnancy in this special time was a stressful life event rather than an event of happiness [29]. It could induce psychological problems and even suicidal ideations and attempts. Another reason may be that women within one year after pregnancy were more likely to experience depression [30, 31], which was a strong cause of suicide among women [19]. The results indicate that the possibility of suicide attempts was 35% higher in participants who were pregnant for the first time at the age of 17 than those at the age of 14 and 15 years. The possible reason was that older adolescents had lower self-compassion as they may be more difficult to believe in the self-compassion message (e.g., I am deserving of kindness), which induced worse emotional well-being and further increased the risk of suicide [32].

Perceived health status was measured in this study, as it was suggested to be a good indicator of the whole health condition [21]. Our results were in line with the previous findings that people with worse health conditions were at elevated risk for mental disorders, including suicide [33, 34]. One study among older adults also found that a negative perception of one’s health status was a significant risk factor for suicide attempts [35]. The current study further supports the relationship in women with adolescent pregnancy that bad health condition was associated with a higher risk of attempting suicide. The possible underlying reasons were that poor health condition resulting in illness burden, functional impairment, and decline in self-care ability, which would increase intentional self-harm behaviors [36, 37]. In addition, perceived poor health may be a proxy for depressive symptoms to some extent, which was a main risk factor of suicide among women [19]. It is better to measure depressive symptoms in future research to clarify the relationship between perceived health and suicide.

Among different dimensions of social support, only the support from friends was found to be associated with suicide attempts in the current study. It is in accordance with the findings of some previous studies. Kuper et al. [38] found that friends' support was negatively associated with suicide attempts of the past year, but the association between family's support and suicide attempts was not significant in the transgender and gender-nonconforming population. Fredrick et al. [39] found that support from friends was a strong buffer between depression and suicidal ideation among adolescent girls than boys. Lacking friends' support may leave young women, especially adolescent girls, feeling alone and isolated. It is consistent with the interpersonal-psychological theory of suicide, which suggests that suicide attempts are associated with a shortage of support and isolation, especially when paired with stressful experiences (e.g., pregnancy during adolescence) [40].

Results of the current study should be interpreted within the context of several limitations. First, our study does not address the causality of the relationships between suicide attempts and related factors since it was a cross-sectional survey. However, socio-demographic factors were relatively stable and pregnancy-related factors were retrospectively assessed, which can be considered as factors existing before the suicide attempt. Second, survival bias may exist which underestimated the prevalence of suicide attempts. Because women with suicide attempt experience finally succeed in suicide was exclude. Third, we only assessed whether the participants experienced suicide attempts in the past 12 months without in-depth information, such as experience of stressful life events for those whose first pregnancy were happened more than one year ago and how many times and the real intention of the suicide attempts. Information on stressful life events should be collected as they could also stimulate suicidal ideation and in-depth information on suicide attempts was helpful for targeted intervention. Qualitative methods could be used to clarify these questions in future research. Fourth, reasons for marriage before 18 were not collected, which may be associated with suicide attempts. Reasons, such as forced marriage may increase the risk of suicide attempts. Instead, the participants were asked if the first pregnancy was planned or unplanned and if the reason was normal pregnancy or abnormal pregnancy (e.g., rape, sexual abuse). Even though abnormal pregnancy is stigmatized in Bangladesh society, we did not find an association between abnormal pregnancy and suicide attempts. It may be because that some participants did not report this stigmatized experience. Government data or medical data on pregnancy caused by rape or sexual abuse should be considered in further research.

## Conclusions

Suicidality represents a major societal and health care problem, it thus should be given a high priority in many realms, particularly in some vulnerable populations. This study suggests that the prevalence of suicide attempts was high in young women with adolescent pregnancy. It also underscores the importance of risk roles of shorter time (especially the first year) after the pregnancy and perceived bad health condition, and the importance of the protective role of friends' support for suicide attempts. The results imply that suicides in young women who were pregnant for the first time at adolescent age may be prevented by providing more support from friends, and perceived health status may be useful as a screening question to identify the risk of suicide in post-natal clinics.

## Data Availability

The data are available from the corresponding author on reasonable request.

## Abbreviations

OR: Odds ratio
CI: Confidence interval

## References

[1] United Nations Population Fund (UNFPA). Motherhood in childhood: facing the challenge of adolescent pregnancy. UNFPA; 2013. https://unfpa.org/sites/default/files/pub-pdf/EN-SWOP2013-final.pdf. Accessed 28 Sep 2019.

[2] Darroch J WV, Bankole A, Ashford LS. Adding it up: Costs and benefits of meeting the contraceptive needs of adolescents. New York: Guttmacher Institute; 2016. https://www.guttmacher.org/report/adding-it-meeting-contraceptive-needs-of-adolescents. Accessed 28 Sep 2019.

[3] Loaiza E, Liang, M. Adolescent Pregnancy: A Review of the Evidence. New York: UNFPA; 2013. https://www.unfpa.org/publications/adolescent-pregnancy. Accessed 28 Sep 2019.

[4] Human Development Indices and Indicators: 2018 Statistical Update. New York: UNDP; 2018. http://www.hdr.undp.org/en/content/human-development-indices-indicators-2018-statistical-update. Accessed 28 Sep 2019.

[5] Wilson-Mitchell K, Bennett J, Stennett R (2014). Psychological health and life experiences of pregnant adolescent mothers in Jamaica. Int J Environ Res Public Health.

[6] Pinheiro RT, da Cunha Coelho FM, da Silva RA, de Avila QL, de Mattos Souza LD, Castelli RD, de Matos MB, Pinheiro KA (2012). Suicidal behavior in pregnant teenagers in southern Brazil: social, obstetric and psychiatric correlates. J Affect Disord.

[7] Taliaferro LA, Muehlenkamp JJ (2014). Risk and protective factors that distinguish adolescents who attempt suicide from those who only consider suicide in the past year. Suicide Life Threat Behav.

[8] DeCou CR, Lynch SM (2018). Sexual orientation, gender, and attempted suicide among adolescent psychiatric inpatients. Psychol Serv.

[9] Bachmann S (2018). Epidemiology of suicide and the psychiatric perspective. Int J Environ Res Public Health.

[10] Yang LS, Zhang ZH, Sun L, Sun YH, Ye DQ (2015). Prevalence of suicide attempts among college students in China: a meta-analysis. PLoS ONE.

[11] Bilsen J (2018). Suicide and youth: risk factors. Front Psychiatry.

[12] Islam MM, Islam MK, Hasan MS, Hossain MB (2017). Adolescent motherhood in Bangladesh: trends and determinants. PLoS ONE.

[13] Sharmin Salam S, Alonge O, Islam MI, Hoque DME, Wadhwaniya S, Ul Baset MK, Mashreky SR, El Arifeen S (2017). The burden of suicide in rural Bangladesh: Magnitude And Risk Factors. Int J Environ Res Public Health.

[14] Phillips JA, Hempstead K (2017). Differences in U.S. suicide rates by educational attainment, 2000–2014. Am J Prev Med..

[15] Elbogen EB, Molloy K, Wagner HR, Kimbrel NA, Beckham JC, Van Male L, Leinbach J, Bradford DW (2020). Psychosocial protective factors and suicidal ideation: Results from a national longitudinal study of veterans. J Affect Disord.

[16] Halim KS, Khondker L, Wahab MA, Nargis F, Khan SI (2010). Various factors of attempted suicide in a selected area of Naogaon district. Mymensingh Med J.

[17] Faisal-Cury A, Menezes PR, Quayle J, Matijasevich A (2017). Unplanned pregnancy and risk of maternal depression: secondary data analysis from a prospective pregnancy cohort. Psychol Health Med.

[18] van de Loo KFE, Vlenterie R, Nikkels SJ, Merkus P, Roukema J, Verhaak CM, Roeleveld N, van Gelder M (2018). Depression and anxiety during pregnancy: the influence of maternal characteristics. Birth.

[19] Indu PS, Anilkumar TV, Pisharody R, Russell PSS, Raju D, Sarma PS, Remadevi S, Amma K, Sheelamoni A, Andrade C (2017). Prevalence of depression and past suicide attempt in primary care. Asian J Psychiatr.

[20] Zadravec Sedivy N, Podlogar T, Kerr DCR, De Leo D (2017). Community social support as a protective factor against suicide: a gender-specific ecological study of 75 regions of 23 European countries. Health Place.

[21] Miilunpalo S, Vuori I, Oja P, Pasanen M, Urponen H (1997). Self-rated health status as a health measure: the predictive value of self-reported health status on the use of physician services and on mortality in the working-age population. J Clin Epidemiol.

[22] National Institute of Mental Health. Ask Suicide-Screening Questions (ASQ) Toolkit. https://www.nimh.nih.gov/research/research-conducted-at-nimh/asq-toolkit-materials/index.shtml#outpatient. Accessed 2 Jul 2018.

[23] Zimet GD, Powell SS, Farley GK, Werkman S, Berkoff KA (1990). Psychometric characteristics of the Multidimensional Scale of Perceived Social Support. J Pers Assess.

[24] Meehan PJ, Lamb JA, Saltzman LE, O'Carroll PW (1992). Attempted suicide among young adults: progress toward a meaningful estimate of prevalence. Am J Psychiatry.

[25] Feroz A, Islam S, Reza S, Rahman A, Sen J, Mowla M, Rahman M (2012). A community survey on the prevalence of suicidal attempts and deaths in a selected rural area of Bangladesh. J Med.

[26] Naved RT, Akhtar N (2008). Spousal violence against women and suicidal ideation in Bangladesh. Womens Health Issues.

[27] Nock MK, Borges G, Bromet EJ, Alonso J, Angermeyer M, Beautrais A, Bruffaerts R, Chiu WT, de Girolamo G, Gluzman S (2008). Cross-national prevalence and risk factors for suicidal ideation, plans and attempts. Br J Psychiatry.

[28] World Health Organization. Maternal, newborn, child and adolescent health. https://www.who.int/maternal_child_adolescent/topics/adolescence/development/en/. Accessed 28 Sep 2019.

[29] Torres R, Goyal D, Burke-Aaronson AC, Gay CL, Lee KA (2017). Patterns of symptoms of perinatal depression and stress in late adolescent and young adult mothers. J Obstet Gynecol Neonatal Nurs.

[30] Putnam KT, Wilcox M, Robertson-Blackmore E, Sharkey K, Bergink V, Munk-Olsen T, Deligiannidis KM, Payne J, Altemus M, Newport J (2017). Clinical phenotypes of perinatal depression and time of symptom onset: analysis of data from an international consortium. Lancet Psychiatry.

[31] Gavin NI, Gaynes BN, Lohr KN, Meltzer-Brody S, Gartlehner G, Swinson T (2005). Perinatal depression: a systematic review of prevalence and incidence. Obstet Gynecol.

[32] Bluth K, Campo RA, Futch WS, Gaylord SA (2017). Age and gender differences in the associations of self-compassion and emotional well-being in a large adolescent sample. J Youth Adolesc.

[33] Ferro MA (2016). Major depressive disorder, suicidal behaviour, bipolar disorder, and generalised anxiety disorder among emerging adults with and without chronic health conditions. Epidemiol Psychiatr Sci.

[34] Black J, Bond MA, Hawkins R, Black E (2019). Test of a clinical model of poor physical health and suicide: the role of depression, psychosocial stress, interpersonal conflict, and panic. J Affect Disord.

[35] Lee H, Seol KH, Kim JW (2018). Age and sex-related differences in risk factors for elderly suicide: differentiating between suicide ideation and attempts. Int J Geriatr Psychiatry.

[36] Ahmedani BK, Peterson EL, Hu Y, Rossom RC, Lynch F, Lu CY, Waitzfelder BE, Owen-Smith AA, Hubley S, Prabhakar D (2017). Major physical health conditions and risk of suicide. Am J Prev Med.

[37] Conwell Y, Duberstein PR, Hirsch JK, Conner KR, Eberly S, Caine ED (2010). Health status and suicide in the second half of life. Int J Geriatr Psychiatry.

[38] Kuper LE, Adams N, Mustanski BS (2018). Exploring cross-sectional predictors of suicide ideation, attempt, and risk in a large online sample of transgender and gender nonconforming youth and young adults. LGBT Health.

[39] Fredrick SS, Demaray MK, Malecki CK, Dorio NB (2018). Can social support buffer the association between depression and suicidal ideation in adolescent boys and girls?. Psychol Sch.

[40] Van Orden KA, Witte TK, Cukrowicz KC, Braithwaite SR, Selby EA, Joiner TE (2010). The interpersonal theory of suicide. Psychol Rev.

